# Soft-Shelled Turtle Peptide Supplementation Modifies Energy Metabolism and Oxidative Stress, Enhances Exercise Endurance, and Decreases Physical Fatigue in Mice

**DOI:** 10.3390/foods11040600

**Published:** 2022-02-19

**Authors:** Hao Zhong, Jinyuan Shi, Junhui Zhang, Qianqian Wang, Yipeng Zhang, Peng Yu, Rongfa Guan, Fengqin Feng

**Affiliations:** 1 College of Food Science and Technology, Zhejiang University of Technology, Hangzhou 310014, China; zhonghao@zjut.edu.cn; 2 College of Biosystems Engineering and Food Science, Zhejiang University, Hangzhou 310058, China; shijinyuan_lydia@163.com (J.S.); zhangjunhui9916@163.com (J.Z.); w5982234537@163.com (Q.W.); 12113008@zju.edu.cn (Y.Z.); 3 Yuyao Lengjiang Turtle Industry, Ningbo 315400, China; simba90@163.com

**Keywords:** soft-shelled turtle peptide, antifatigue, oxidative stress, NRF2, KEAP1

## Abstract

The potential of soft-shelled turtle peptides (STP) against fatigue was evaluated. Mice orally supplemented with STP significantly increased the swimming time until tiredness by 35.4–57.1%. Although not statistically significant, STP increased muscle and thymus mass. In addition, the serum lactate, ammonia, blood urea nitrogen content and creatine kinase activity in STP-fed mice were dramatically decreased when compared to the control group. Furthermore, STP supplementation increased the reserves of liver glycogen and muscle glycogen, thus improved the energy metabolism system of mice. STP treatment contributed to increased superoxide dismutase (SOD) and glutathione peroxidase (GSH-Px) activities as well as a decrease in malondialdehyde (MDA), indicating an improvement in oxidative stress protection. The Western blot (WB) results indicated that the STP supplement effectively altered the expression of oxidative stress-related protein by modulating the NRF2/KEAP1 pathway. In summary, STP affected NRF2/KEAP1 levels in skeletal muscle, leading to antioxidant activity and a slower time to exhaustion during exercise.

## 1. Introduction

Fatigue is a symptom of sub-health state and can be classified as physical or mental exhaustion according to the cause [1]. Physical fatigue is usually shown in a deterioration in exercise performance and even difficulty in sustaining regular physical activities [2]. There are many reasons underlying exercise-induced fatigue: first, the consumption of energy sources and the over-production and accumulation of metabolic products [3,4]; second, the excessive generation of reactive oxygen species (ROS) and the disorder of immune system [5]; last, the blood-oxygen concentration balance as well as muscle and liver glycogen homeostasis are difficult to maintain [6]. Excessive fatigue and lack of reasonable adjustment have been a threat to human health for many years [7,8,9]. Therefore, researchers have tended to focus on searching for natural antifatigue substances expecting to boost athletic performance and eliminate exhaustion in human beings [10].

Recently, bioactive peptides derived from food proteins have been discovered with potent physiological activities such as antioxidant [11], anticancer, antibacterial, antifungal, and antitumor properties, as well as ion channel blockers, which opened a new standpoint for developing fatigue-relieving products [12]. Researchers have successfully isolated peptides possessing antifatigue potentials from aquatic organisms, e.g., sea cucumber, cod, hairtail and turtle [13,14,15,16]. The soft-shelled turtle (*Pelodiscus sinensis*) is a traditional Chinese nourishing food. It has also been considered as a dietary remedy for physical and mental fatigue since ancient times. With the in-depth research on the components of this turtle, previous studies found that soft-shelled turtle peptides performed well in in vitro antioxidant and anticancer activities [17,18]. However, there are few reports exploring the antioxidant effects in vivo and the mechanisms of its antifatigue activity.

Hypotheses have been invoked to clarify the mechanism of exercise fatigue, among which three theories, clogging, exhaustion, and radical are the more widely researched [19]. According to the clogging theory, metabolites such as blood urea nitrogen (BUN) and blood lactate (LA) might accumulate during exercise, resulting in excessive metabolite accumulation and a reduction in muscle capacity [20]. The exhaustion theory proposed that the low utilization and depletion of endogenous energy such as glucose and liver glycogen resulted in physical fatigue [21]. The radical theory emphasizes that strenuous activity can cause extravagant production of free radicals in skeletal muscle, resulting in fatigue [22]. Current studies pointed out that bioactive peptides from aquatic products fought against fatigue through eliminating the aforementioned metabolites, reducing oxidative damage or improving glycogen metabolism [19,23,24]. However, different bioactive peptides have diverse functional properties, and thus further studies need to be carried out to explore the antifatigue mechanisms.

The purpose of this research was to assess the antifatigue effects of soft-shelled turtle peptides (STP) in mice, and the biological activity of STP was analyzed through the exhaustive swimming model. Furthermore, the underlying mechanism of STP on physical fatigue was investigated through determining the following parameters: in vivo antioxidant indicators such as the content of malondialdehyde (MDA), the activity of superoxide dismutase (SOD) and glutathione peroxidase (GSH-Px); the biochemical indexes such as lactate (LA), ammonia (NH3), and blood urea nitrogen (BUN) content; the lactate dehydrogenase (LDH) activity; the creatine kinase (CK) activity and blood glucose (Glu) level in the serum; liver glycogen (LG) and muscle glycogen (MG) level in the gastrocnemius; and the expression of the NRF2/KEAP1 protein in skeletal muscle of mice.

## 2. Materials and Methods

### 2.1. Materials

STPs were generated from soft-shelled turtle (*Pelodiscus sinensis*) protein hydrolysates, which were gratefully offered by Hangzhou Kangyuan Food Science and Technology Co., Ltd. (Hangzhou, China) as a gift, and kept at −80 °C. The basic chemical components, amino acid content, and molecular mass distribution of STPs are shown in Tables S1–S3, respectively.

### 2.2. Animal Experiment Protocol

The animal experiment was designed as described in our previous study with some modifications [25]. Male ICR mice (weight 20 ± 2 g) were purchased from SLAC Laboratory Animal Co., Ltd.(Shanghai, China) and subsequently maintained at Animal Experimental Center of Zhejiang Chinese Medicine University. Ambient conditions were maintained at 25 ± 2 °C, 50 ± 5% relative humidity, and a 12/12 h light–dark cycle. After a week of adaptation, they were randomly divided into the following 5 groups (n = 18): (1) normal control group (distilled water only, control), (2) whey peptides group (0.5 mg g^−1^ BW, WP), (3) low-dose STP group (0.25 mg g^−1^ BW, STP-L), (4) medium-dose STP group (0.5 mg g^−1^ BW, STP-M), and (5) high-dose STP group (1.0 mg g^−1^ BW, STP-H). Each group was given the appropriate sample by daily gavage administration for 30 days [25].

### 2.3. Body Weight and Organ Index

The body weight and organ index were record and analyzed according to previous study [25]. The mice were weighed weekly. At the end of the experiment, the mice were dissected, and the liver, leg muscle, kidney, spleen and thymus were taken and rinsed with normal saline, and the water was absorbed by filter paper. The weight of the liver was accurately weighed. Part of the liver was placed in the fixed solution for histological observation, and the rest of the tissues were stored in the refrigerator at −80 °C. The equation for determining organ index is provided below:

(1)  Organ index (%)=Organ weight (g)/Body weight (g)×100

### 2.4. Histological Observation

The liver samples were quickly immobilized in 10% formalin for 24 h, then dehydrated in a series of gradient ethanol doses of 70% to 100%, and embedded in paraffin using tissue embedding techniques. The sample was subsequently sliced into 4 μm pieces and stained with hematoxylin and eosin using a slicer. An optical microscope with a camera (Nikon Eclipse CI, Tokyo, Japan) was employed to take photomicrographs.

### 2.5. Weight-Loaded Forced Swimming Test

After 30 min of the final gavage administration, 10 mice were randomly selected from each group for the weight-loaded forced swimming test, which was in accordance with the assay of Hu et al. with some modifications [26]. Each mouse was washed with soap to remove the oil on the body surface first, and then a lead sheath (5% of each mouse’s body weight) was loaded on the root of the tail and the mice were dropped individually into a swimming pool (depth 40 cm, water temperature 25 °C). The exhausting swimming time was determined when the mouse sank to the bottom and could not rise to the surface of liquid in 10 s.

### 2.6. Forelimb Grip Strength Test

After 30 min of the final gavage administration, 10 mice were randomly selected from each group for the forelimb grip strength test. Briefly, when the mouse grasped the available grids with the front paw, the tail was lifted and the mouse gently pulled back parallel to the grids, and the maximum force figure would immediately appear on the low-force testing system (Model-RX-5, Aikoh Engineering, Nagoya, Japan) [27]. The average value of three replicates was the final force value of the mouse’s forelimb grip strength.

### 2.7. Determination of Biochemical Indicators

After 30-day feeding, all mice were compelled to swim for 30 min (water temperature 25 °C) without any weight loaded and then allowed to rest for 30 min. The blood sample was collected from the eyeball and centrifuged at 2800× *g* and 4 °C for 15 min to obtain serum samples. For further investigation, the liver and gastrocnemius samples were separately immersed in 10% formalin or preserved at −80 °C. Lactate (LA), BUN, NH3, LDH and CK levels in the serum, glycogen in the liver and muscle, liver MDA, SOD and GSH-Px were determined according to the kit instructions (Nanjing Jiancheng Bioengineering Institute, Nanjing, China).

### 2.8. Western Blot Analysis

The Western blot assay was performed according to our previous study [28]. Total protein from gastrocnemius samples (~0.1 g) were prepared by using lysis buffer (Thermo Pierce, Rockford, IL, USA) along with phosphatase inhibitor on ice, and then centrifuged at 6000× *g* for 4 min to obtain the supernatant. The protein concentration was determined by the method of bicinchoninic acid (Bio-Rad, Hercules, CA, USA). SDS-PAGE was used to separate proteins (NRF2, KEAP1 and HO-1), which were then loaded to polyvinylidene difluoride (PVDF, Beyotime, Shanghai, China) membranes. Membranes were treated with the primary antibody (Abcam, Cambridge, UK) overnight at 4 °C, then washed and incubated with the secondary antibodies for 30 min at room temperature. The signal was detected using Gel Image Analysis System (Tanon-3500R, Shanghai, China).

### 2.9. Statistical Analysis

The experimental results were analyzed employing SPSS 20.0 (IBM Corp., New York, NY, USA) and given as mean ± standard deviation (SD) values of triplicates. The statistical significance (*p* < 0.05) was determined using one-way analysis of variance (ANOVA) according to the Tukey’s test.

## 3. Results

This section is by subheadings. It provides a concise and precise description of the experimental results, their interpretation, as well as the experimental conclusions that can be drawn.

### 3.1. STP Augment Physiological Indexes in Mice

According to the findings, there were no significant changes in body weight, food and energy consumption, and organ index among the five groups (*p* > 0.05). The body weight of all groups increased during 30 days of intragastric administration, which demonstrated that the growth of mice was in a natural state (Table S4). As shown in Table 1, compared to the control group, the muscle content, the spleen index and the thymus index in WP group and STP groups increased. The implications demonstrated that WP and STP could relieve the fatigue symptoms of mice and improve the immune ability of mice to a certain extent.

### 3.2. STP Would Not Affect Morphological and Pathological Changes

**Figure 1:**
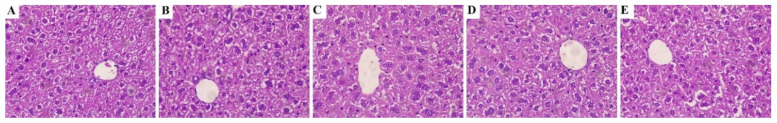
**Effects of STP on histological changes in liver tissue.** (H&E, magnification: 400×) (**A**) Control; (**B**) WP; (**C**) STP-L; (**D**) STP-M; (**E**) STP-H.

The histological changes of mice were observed. As shown in Figure 1, in comparison with the control group, the hepatic lobule structure was normal in all experimental groups, the configuration of the hepatic sinuses and cords did not alter considerably, and the size of hepatic cells was consistent. These results indicated that STP would not cause histopathological changes in livers of mice.

### 3.3. STP Could Improve Exercise Tolerance

**Figure 2:**
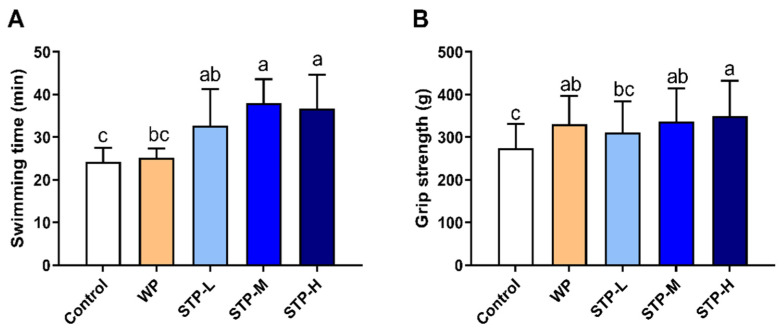
**Effects of STP on exercise tolerance in mice.** (**A**) Exhaustive swimming time of mice; (**B**) forelimb grip strength of mice. Means followed by different letters are significantly different (*n* = 8, *p* < 0.05).

The weight-loaded forced swimming test and forelimb grip strength test were performed to assess the impact of STP on exercise tolerance in mice. As depicted in Figure 2A, the swimming times to exhaustion in all the STP groups were significantly longer than the control group. Furthermore, the STP-M and STP-H groups had obviously prolonged swimming times compared to the WP group. Figure 2B shows that the forelimb grip strength of the STP-M and STP-H groups was considerably higher than that of the control group in a dose-dependent manner. In short, these experiments demonstrated that STP effectively relieved fatigue.

### 3.4. STP Modified Metabolism Accumulation

**Figure 3:**
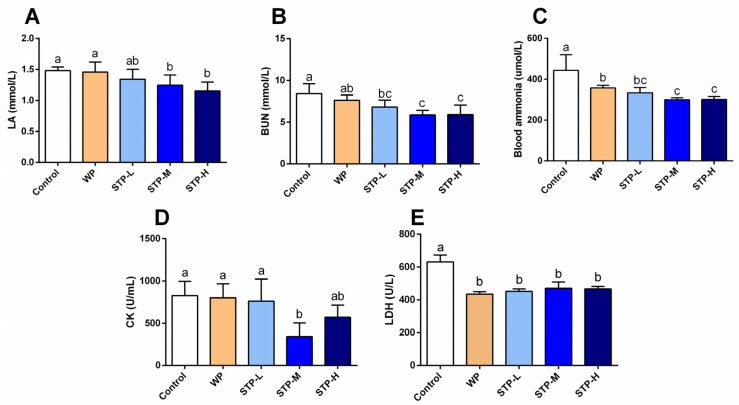
**Effect of the STP on metabolites and muscle protection in mice.** (**A**) LA; (**B**) BUN; (**C**) blood ammonia, (**D**) CK activity, (**E**) LDH activity. Means followed by different letters are significantly different (*n* = 8, *p* < 0.05).

Several biochemical indices, including LA, BUN and blood ammonia (NH3), CK and LDH activities, were analyzed to determine the impact of STP on fatigue alleviation. As shown in Figure 3A–E, compared to the control group, the LA content in STP-M and STP-H groups, and the BUN, ammonia and LDH levels in the STP-L, STP-M and STP-H groups were significantly reduced (*p* < 0.05). In addition, the serum CK activity in the STP-M group was significantly decreased when compared to the control group (*p* < 0.05), with a rate of 58.6%.

### 3.5. STP Increased Energy Storage in Mice

**Figure 4:**
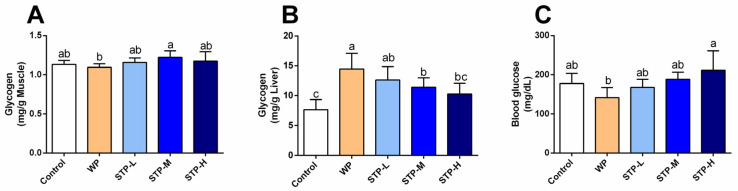
**Effect of STP on energy storage in mice.** (**A**) Muscle glycogen content; (**B**) liver glycogen content; (**C**) blood glucose level. Means followed by different letters are significantly different (*n* = 8, *p* < 0.05).

Glycogen in muscle and liver are main forms of energy storage in the body and are important indicators of fatigue resistance [29]. As shown in Figure 4, the liver glycogen contents in STP-L and STP-M groups were significantly higher than that of control, which were increased by 63.6% and 48.1%. However, the muscle glycogen and blood glucose contents showed that there was no statistically significant difference between the five groups, while the muscle glycogen content increased slightly in the STP-M group and the blood glucose content increased by 19.4% in comparison to the control group. These findings suggested that STP could increase the reserves of liver glycogen and muscle glycogen, improve the energy metabolism system of mice, and enhance the ability of endurance to exercise.

### 3.6. STP Exerted Antioxidant Activity

**Figure 5:**
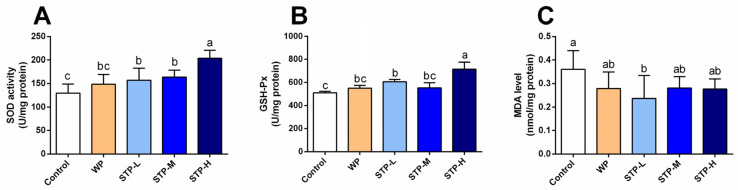
**Effect of STP on antioxidant activity in mice.** (**A**) SOD activity; (**B**) GSH-Px activity; (**C**) MDA content. Means followed by different letters are significantly different (*n* = 8, *p* < 0.05).

It is well known that being overly fatigued is mainly attributed to the over-generation of free radicals and the negative effects of oxidative stress [2]. Thus, several critical oxidation indictors were also examined, such as SOD and GSH-Px activities and MDA level in the serum and gastrocnemius tissue. Compared to the control group, the SOD activity in all STP-treated groups was greatly elevated by 21.3%, 26.5% and 57.1% (Figure 5, *p* < 0.05). Additionally, the GSH-Px activity of all STP groups was higher than the control group, among which the STP-L and STP-H groups showed significant differences (*p* < 0.05) with an increasing rate of 19.0% and 40.4%. The level of MDA was significantly decreased in the STP-L group (*p* < 0.05) in comparison to the control group. Data showed that STP could alleviate fatigue through increasing antioxidant capacity.

### 3.7. STP Modulated the NRF2/KEAP1 Pathway

**Figure 6:**
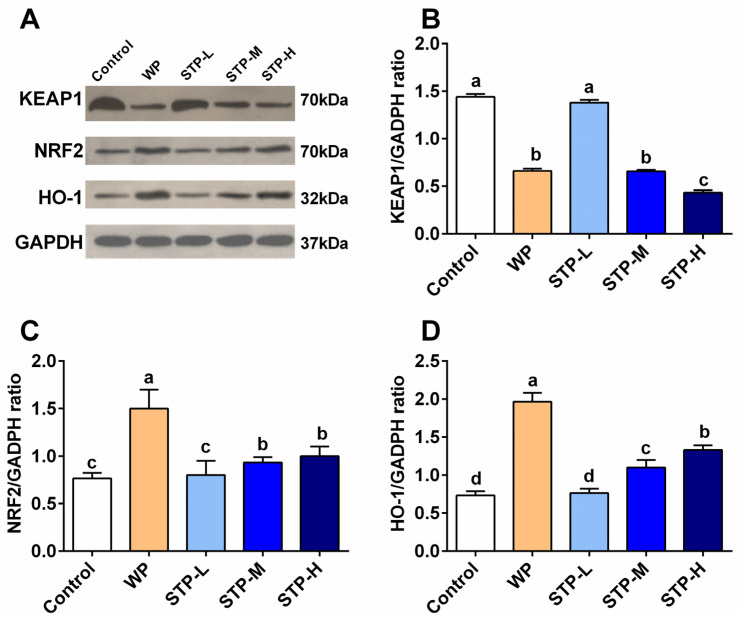
**Effect of STP on NRF2/KEAP1 pathway relative protein in skeletal muscle of mice.** (**A**) Protein expression levels of NRF2, KEAP1 and HO-1, (**B**) KEAP1, (**C**) NRF2, (**D**) HO-1. Means followed by different letters are significantly different (*p* < 0.05).

Nuclear factor (erythroid-derived 2)-related factor 2 (NRF2), together with its suppressive binding partner Kelch-like ECH-associated protein 1 (KEAP1), plays an important role in regulating cellular antioxidant response [30]. The effect of STP on activating the NRF2/ARE signaling pathway was studied in skeletal muscle of mice. As shown in Figure 6B, the expression of KEAP1 protein was significantly downregulated in both STP-M and STP-H groups compared to the control group (*p* < 0.05). As shown in Figure 6C, when compared with the control group, NRF2 expression in STP-M and STP-H groups was significantly upregulated (*p* < 0.05), as it increased by 21.7% and 30.4%. As shown in Figure 6D, the expression of HO-1 in STP-M and STP-H groups was significantly upregulated (*p* < 0.05), which was increased by 21.2% and 81.8%. In summary, there were no distinct differences between the STP-L and control groups, which may be attributed to the low dosage of STP and the maximum upregulation effect of STP-H, indicating that STP can improve the expression of NRF2 and HO-1 proteins in a dose-dependent manner. Combined with the results of the antioxidant test, the MDA level in the STP group decreased, and SOD and GSH-Px activity increased, which can comprehensively prove that STP can improve the antioxidant capacity of mice and reduce oxidative stress and fatigue by modulating the expressions of KEAP1, NRF2 and HO-1 in the NRF2/ARE signaling pathway.

## 4. Discussion

STP is a soft-shell turtle protein hydrolysate that is preferred for bioavailability, taste, and a variety of physiological benefits. Furthermore, peptides can increase NO generation and provide consumers with rapid energy. As a result, when compared to soft-shell turtle protein, STP is a better sports supplement for improving athletic performance. Some studies have reported that loach peptides and mackerel peptides could elevate the endurance capacity and facilitate recovery from fatigue [4,20]. In this work, the antifatigue potential of STP was first investigated in mice. The findings suggest that long-term STP consumption improves exercise performance.

Bioactive peptide supplements are thought to be an efficient tactic for improving exercise tolerance in the field of athletic physiology. The main reason for this is that bioactive peptides have a significant impact on cell metabolism. Bioactive peptides have recently been shown to be effective in lowering KEAP1 and NRF2 expression [25]. The NRF2/KEAP1 axis functions as a ‘thiol-driven master switch’ for ‘system-wide oxidative stress responses’. NRF2 regulates the expression of four major genes involved in the generation of NADPH, a key cofactor that supports antioxidant processes [31]. According to the results of this study, the downregulation of NRF2/KEAP1 in skeletal muscle by STP is linked to improved exercise performance and antioxidant activities. Consistent with a previous in vitro study, Wang et al. found the peptides from soft-shelled turtle enhanced NRF2 level and downregulated KEAP1 [32].

Lactate is created when the supply of oxygen is insufficient during severe exercise [33], and the accumulation of blood serum LA is an important cause of fatigue. With intense exercise, O_2_ and pyruvic acid are reduced by LDH to LA, which decreases the pH, affecting both the cardio-circulating system and skeletal muscle system function. Hydrogen ions (H^+^) separate from lactate and accumulate in the muscles, resulting in tiredness and a reduction in muscular strength and activation. The blood lactate level after exercise is determined by the ratio of lactate creation to lactate removal. Furthermore, hypoglycemia (low blood glucose) during exercise induces exhaustion and poor energy, causing the exercise to be stopped [34]. After swimming with the identical weight, the STP-treated mice showed decreased blood lactate levels (Figure 3A) and greater glucose levels (Figure 4C). When compared to the control group, STP considerably prolonged the time it took to become exhausted during swimming (Figure 2A). The findings indicate that the STP supplement is responsible for the significant reduction of lactate generation and/or elevated lactate metabolism after exercise. Similarly, a previous study reported that the *Hippocampus* peptide improved exercise tolerance by reducing accumulation of lactic acid [35].

When muscular glycogen reserves are depleted, the rate of ATP formation drops and the capacity to exercise is compromised [36]. In general, higher muscle glycogen accumulation capacity may have a direct role in improving exercise endurance and reducing physical exhaustion, as seen by the improved physical performance in mice of the STP-H group. Additionally, the benefits of STP intervention on the alleviation of muscle injury were studied, and it was discovered that STP greatly decreased LDH and CK levels, indicating that STP has the capability to protect muscle from injury during swimming, which is line with the observations of a recent study [25]. According to these biochemical parameters, the STP supplement outscored the WP treatment in terms of metabolite accumulation, muscle damage, and glycogen reserve in the muscles.

Strenuous exercise causes a large energy expenditure, which is followed by the production of excessive free radicals in skeletal muscle, resulting in a state of unbalance between the oxidation and antioxidation functions [37]. As a result, fatigue is directly related to oxidative stress. Therefore, to confirm whether STP has an effect on oxidative systems, the primary antioxidant enzymes as well as the key lipid peroxidation chemicals (MDA) were assessed. The consequences of this research revealed that STP supplement successfully enhanced SOD and GSH-Px activities while decreasing MDA levels, confirming STP exhibited antioxidant capability by maintaining a balance between oxidation and antioxidation mechanisms. According to previous research, peptide supplement increased the body’s antioxidant capacity by supplying hydrogen atoms or electrons that contribute to the clearance of free radical reactions, chelation of metal ions, suppression of the production of ROS, and upregulation of antioxidant enzymes [38].

Furthermore, STP not only modulated the antioxidant systems, but also regulated the protein transcriptional activity in the NRF2 pathway. NRF2, one of the most important regulators of redox reactions in cells, is found in the cytoplasm and interacts with KEAP1 [31]. When cells acquire ROS, NRF2 detaches from KEAP1 and translocates into the nucleus, where it forms a new bond with antioxidant response elements (ARE), whose expression is controlled by the activator of phase enzyme genes such as HO-1 [31]. Typically, NRF2 expression in cells is insufficient to entirely prevent the organism from experiencing oxidative stress during exercise. Thus, STP has the capability to augment the upregulation transcription of NRF2 and hence increase the generation of substantial amounts of antioxidants. Therefore, it is clear that the increase in antioxidation systems of STP-supplemented mice can be attributed to the NRF2/KEAP1 regulation.

## 5. Conclusions

STP supplementation before exercise downregulates NRF2/KEAP1 transcription in skeletal muscle, leading to a remarkable improvement in antioxidant activity and muscle mass. STP-supplemented mice also had an extended average duration before becoming weary during exercise, but the efficacy of this benefit requires to be confirmed in a human study. Further experiments would aim to identify the main compounds in STP which contribute to the increased muscle mass and improved exercise endurance. Nevertheless, the present research convinced that STP supplement has a significant enhancement in exercise endurance, increased energy storage and antioxidant activity, and therefore may be used to enhance sport performance or antifatigue activity.

## Figures and Tables

**Table 1:** Mice organ indexes.

Group	Liver (%)	Muscle (%)	Kidney (%)	Spleen (%)	Thymus (%)
Control	5.57± 0.31	5.08 ± 0.93	1.51 ± 0.16	0.29 ±0.05	0.11 ± 0.03
WP	5.86 ± 0.50	5.32 ± 1.00	1.52 ± 0.14	0.31 ± 0.05	0.14 ± 0.04
STP-L	5.57 ± 0.57	5.30 ± 1.00	1.44 ± 0.29	0.32 ± 0.04	0.12 ± 0.05
STP-M	5.43 ± 0.25	5.14 ± 0.98	1.51 ± 0.07	0.27 ± 0.04	0.15 ± 0.06
STP-H	5.20 ± 0.81	5.65 ± 1.06	1.36 ± 0.16	0.30 ± 0.07	0.14 ± 0.03

Note: Muscle weight is the sum of left and right leg muscles.

## Data Availability

Data is contained within the article.

## Supplementary Materials

The following supporting information can be downloaded at: https://www.mdpi.com/article/10.3390/foods11040600/s1. Figure S1: Approval of local ethical commission for animal experiment; Table S1: The basic components of STP; Table S2: Amino acid composition of the STP; Table S3: Molecular mass distribution of STP; Table S4: Mice weigh and energy intake.
