# Supplementary material for: Soft-Shelled Turtle Peptide Supplementation Modifies Energy Metabolism and Oxidative Stress, Enhances Exercise Endurance, and Decreases Physical Fatigue in Mice
Source: Foods. 2022 Feb 19;11(4):600. doi: 10.3390/foods11040600 (PMC8871340; doi:10.3390/foods11040600)
Supplement: Supplementary file 1 [file foods-11-00600-s001.zip › foods-1582899-supplementary.pdf]

## Table S1. The basic components of STP

| | Moisture (%) | Ash (%) | Total protein (%) | Peptide content (%) |
|---|---|---|---|---|
| STP | 4.92±0.06 | 1.32±0.05 | 90.84±0.45 | 87.90±0.08 |

## Table S2. Amino acid composition of the STP

| Amino acids | Content (mg/g) | Ratio (%) |
|---|---|---|
| Aspartic acid | 59.81 | 7.86 |
| Glutamic acid | 115.02 | 15.11 |
| Serine | 23.72 | 3.12 |
| Glycine | 119.99 | 15.76 |
| Histidine | 9.18 | 1.21 |
| Arginine | 31.28 | 4.11 |
| Threonine* | 22.84 | 3.00 |
| Alanine | 56.15 | 7.38 |
| Proline | 65.96 | 8.67 |
| Tyrosine | 21.14 | 2.78 |
| Valine* | 35.00 | 4.60 |
| Methionine* | 14.89 | 1.96 |
| Cystine | 12.22 | 1.61 |
| Isoleucine* | 50.61 | 6.65 |
| Leucine* | 49.09 | 6.45 |
| Phenylalanine* | 23.68 | 3.11 |
| Lysine* | 50.59 | 6.65 |
| Tryptophan | ND | ND |
| Essential amino acids | 246.67 | 32.41 |
| Total amino acids | 761.17 | 100 |

"*" represents essential amino acids, "ND" represents not detected.

## Table S3 Molecular mass distribution of STP

| Molecular mass (Da) | Retention time (min) | Ratio of Area/(%) |
|---|---|---|
| 3000-5000 | 14.49-15.41 | 0.11 |
| 2000-3000 | 15.41-16.98 | 0.60 |
| 1000-2000 | 16.98-18.31 | 6.06 |
| 500-1000 | 18.31-19.86 | 23.68 |
| 180-500 | 19.86-20.47 | 56.05 |
| <180 | 20.47 | 13.50 |

**Table S4.** Mice weigh and energy intake

| Group | Body weight(g) | | | Food intake (g/d) | Energy intake (g/d) |
|---|---|---|---|---|---|
| | Initial | Terminal | Increased | | |
| Control | 26.02±1.46 | 36.01±3.44 | 9.99±2.81 | 4.72±0.22 | 17.45±0.80 |
| WP | 26.10±1.46 | 37.19±2.92 | 11.09±2.41 | 4.85±0.37 | 17.95±1.37 |
| STP-L | 25.99±1.22 | 35.74±2.37 | 9.74±2.40 | 4.67±0.35 | 17.30±1.30 |
| STP-M | 25.78±1.16 | 36.51±2.53 | 10.73±2.49 | 4.66±0.12 | 17.23±0.45 |
| STP-H | 25.94±1.15 | 35.93±2.69 | 9.99±2.14 | 4.71±0.18 | 17.44±0.66 |

# 动物实验伦理审查同意书

**Affidavit of Approval of Animal Ethical and Welfare**

| 伦理申请编号 | 201909-0450 | 批准编号 Approval No. | IACUC-20190930-07 |
|---|---|---|---|

本《动物实验方案》经过实验动物管理与伦理委员会审核，符合动物保护、动物福利和伦理原则，符合国家实验动物福利伦理的相关规定。方案的相关信息如下：

The Animal experiment protocol listed below has been reviewed and approved by Laboratory animal management and ethics committee of ZCMU.

| | | | | | |
|---|---|---|---|---|---|
| 实验名称 Protocol Title | 甲鱼肽的抗疲劳作用<br>Anti-fatigue function of soft-shelled turtle peptides in mice | | | | |
| 申请人姓名 Applicant | 史晋源<br>Shi Jinyuan | 职称/学位 Title/Degree | 硕士<br>master | 邮箱 Email | 970354908@qq.com |
| 实验负责人 Principal Investigator | 冯凤琴<br>Feng Fengqin | 职称/学位 Title/Degree | 教授<br>professor | 邮箱 Email | fengfq@zju.edu.cn |
| 院系（部门） Department | 浙江大学生物系统与食品科学学院<br>college of biosystems engineering and food science, zhejiang university | | | | |
| 拟实验时间 Period of Protocol | 2019-11-01 - 2019-12-31 | 实验动物使用许可证 Number of Animal Use Permit | | SYXK（浙）2018-0012 | |
| 审核意见 Results of Inspection | □符合动物福利伦理要求，可以进行实验。 Agree<br>□调整方案后，可以进行实验。 Agree after modify | | | | |

浙江中医药大学实验动物管理与伦理委员会
Animal Ethical and Welfare Committee of ZCMU

中心IACUC主席（Chairman）：

日期(Date)：

Figure S1 Approval of local ethical commission for animal experiment
